# Institutional Governance for Sustainable Utilisation of Healthcare IoT Technologies: Moving Beyond Technology Acceptance to Conditions of Use

**DOI:** 10.3390/healthcare14091225

**Published:** 2026-05-02

**Authors:** Yuyao Lang, Aini Aman, Kamarul Baraini Keliwon, Syaima Adznan, Hui Zhang

**Affiliations:** Faculty of Economics and Management, University Kebangsaan Malaysia (UKM), Bangi 43600, Selangor, Malaysia; p113373@siswa.ukm.edu.my (Y.L.); baraini@ukm.edu.my (K.B.K.); syaima@ukm.edu.my (S.A.); p113462@siswa.ukm.edu.my (H.Z.)

**Keywords:** institutional governance, technology utilisation, healthcare, regulatory compliance, organisational support, Internet of Things (IoT), sustainable digital transformation

## Abstract

Background/Objectives: The digital transformation of healthcare has become a key component of building resilient and sustainable health systems. However, the long-term sustainability of digital health technologies depends not only on user acceptance but also on the institutional governance conditions that shape how these technologies are implemented and utilised in practice. This study examines how institutional factors shape the sustainable utilisation patterns of Internet of Things (IoT) technologies in regulated healthcare environments, with hospital IoT-based asset management systems, a mature and widely deployed use case in China’s public hospitals, providing the empirical context for the investigation. Methods: Drawing on institutional theory and the Technology Acceptance Model (TAM), we conceptualise user perceptions as behavioural micro-foundations through which institutional conditions influence technology utilisation. A survey of 293 healthcare professionals from two large public hospitals in China was analysed using Structural Equation Modelling (SEM), incorporating mediation and Multi-Group Analysis (MGA). Results: The results demonstrate that technical compatibility (TC) significantly enhances perceived ease of use (PEU) (β = 0.40), while organisational support (OS) positively influences both perceived usefulness (PU) (β = 0.35) and PEU (β = 0.30). Conversely, regulatory compliance (RC) negatively affects PU (β = −0.25) and PEU (β = −0.20), revealing a tension between accountability requirements and operational efficiency. The model explains between 58% and 67% of the variance in key constructs. Conclusions: Overall, the findings indicate that sustainable utilisation patterns depend on alignment between technological capabilities and institutional governance conditions, with user perceptions operating as behavioural micro-foundations through which institutional effects are transmitted. By integrating institutional theory with technology acceptance research, this study contributes a governance perspective for understanding sustainable digital transformation in healthcare systems and provides practical insights for designing interoperable, compliant, and supportive digital health infrastructures to enhance hospital operational efficiency and quality of care.

## 1. Introduction

The digital transformation of healthcare has emerged as a critical pathway toward the development of more resilient, efficient, and sustainable health systems. As healthcare systems worldwide face increasing pressures from ageing populations, rising medical costs, and growing service demand, digital technologies such as the Internet of Things (IoT) are increasingly deployed to improve operational efficiency, optimise resource utilisation, and enhance service delivery. These technologies play a particularly important role in modern health care management by reducing administrative redundancy, improving patient safety, and alleviating the severe workloads of frontline medical staff [1,2]. Through real-time monitoring, asset tracking, and data-driven coordination of healthcare resources, IoT technologies offer the potential to reduce resource waste, improve equipment utilisation, and strengthen the resilience of healthcare infrastructure [3,4,5,6]. However, despite the considerable technological potential of digital health solutions, their sustainable utilisation remains uneven across healthcare organisations. Many systems are successfully implemented yet remain underutilised or inconsistently used in daily clinical practice [7,8].

In healthcare settings, why do medical professionals actively use existing technology rather than merely accept it at psychological level? Addressing this question requires moving beyond individual-level psychological perspectives to examine the institutional conditions that shape technology-use behaviour [9]. The healthcare environment presents a distinctive dual nature: on the one hand, there is a technology-driven demand for operational efficiency; on the other hand, there are strict constraints shaped by institutional logic. These constraints include rigorous laws and regulations governing data protection and patient privacy, professional accountability structures mandating documentation and compliance, and hierarchical organisational arrangements that allocate resources and define strategic priorities. These institutional factors not only influence technology adoption decisions but also determine whether technologies can be translated into feasible, compliant, and sustainable practices in daily clinical work [10].

For a long time, the dominant paradigm in healthcare technology research has focused on individual acceptance, drawing heavily on the Technology Acceptance Model (TAM) and its extensions (e.g., Unified Theory of Acceptance and Use of Technology (UTAUT)) [11,12], identifying perceived usefulness (PU) and perceived ease of use (PEU) as the determinants of adoption intention [13,14]. However, as the digital transformation of healthcare deepens, this intention-oriented explanatory framework reveals significant limitations [15]. Acceptance perceptions describe users’ psychological cognitions but fail to encapsulate the institutional arrangements that determine whether positive cognitions translate into sustained practices [16,17]. In realistic scenarios, healthcare professionals might perceive an IoT technology as highly valuable clinically, yet find it unworkable in a busy outpatient setting due to cumbersome compliance procedures; alternatively, they might find the system interface user-friendly but have to abandon it due to a lack of support of organisations to integrate the technology into clinical workflows.

The literature on IoT application in the healthcare sector particularly reflects this deficiency. Existing studies have documented the correlation between acceptance factors and adoption intentions [18], but they lack explanatory power regarding how institutional mechanisms empower or constrain subsequent use in the post-adoption phase. Especially after 2020 [19], with the tightening of global data governance regulations, such as the implementation of the Personal Information Protection Law (PIPL) in China [20,21] and the General Data Protection Regulation (GDPR) in the European Union [22], compliance has shifted from a back-office management issue to a front-line operational constraint. This evolution in the institutional environment necessitates shifting our research focus from predicting acceptance to explaining conditions of use.

This study aims to fill this gap by proposing a novel analytical perspective that examines the institutional governance conditions explaining IoT technology use in hospital asset management. Rather than treating acceptance perceptions as the endpoints of analysis, we reposition them as mediating mechanisms, namely the behavioural micro-foundations. Through these micro-foundations, macro-institutional factors translate into micro-utilisation behaviours. This study focuses on three core institutional variables rooted in Scott’s [10] three pillars of institutions: technical compatibility (TC, representing the structural/cognitive pillar), regulatory compliance (RC, representing the regulative pillar), and organisational support (OS, representing the normative pillar).

The recent literature emphasises the urgency of this perspective. A meta-analysis by Veiga et al. [23] pointed out that while performance expectancy is a universal driver globally, the high heterogeneity of findings across regions indicates that theoretical models must be contextualised according to specific regional and institutional backgrounds. Alauddin et al. [24] highlighted in their study on digital platforms that the impact of digital tools is fundamentally reshaped by the degree of workflow integration and labour practices, suggesting that technology cannot be evaluated in isolation from the work structures in which it is embedded. Similarly, Mohaiyadin et al. [25] demonstrated that in regulated industries, accountability and governance structures are the critical determinants of transparency and the success of technology implementation.

The research context provides immense value for examining these relationships. Public hospitals in China operate under a highly centralised governance structure, featuring comprehensive regulatory oversight and established hierarchical arrangements [26]. These institutional characteristics make the effects of RC and OS particularly salient, providing clear conditions for observing institutional influences that might operate more subtly in less regulated environments.

While the theoretical framework developed in this study is intended to apply to healthcare IoT technologies broadly, the empirical analysis is anchored in a specific use case: hospital IoT-based asset management systems, including intelligent infusion monitoring, bedside interactive terminals, and equipment location and utilisation tracking. This bounded empirical focus is a deliberate design choice. Asset management represents the most mature and widely deployed category of healthcare IoT in China’s public hospitals, offering a setting in which institutional conditions have had sufficient time to stabilise and produce observable variation in user perceptions and use behaviours. We return in Section 7 to the question of how confidently these findings can be extrapolated to other IoT applications in healthcare, such as continuous physiological monitoring, tele-intensive-care systems, or patient-worn devices, which operate under distinct regulatory, clinical, and workflow conditions.

## 2. Theoretical Framework and Hypotheses Development

### 2.1. Technology Application as Institutionally Embedded Behaviour

Technology application in healthcare cannot be understood as an individual-level phenomenon independent of its institutional context. Healthcare institutions operate through governance structures that define legitimate actions, allocate resources, and impose accountability requirements. These structures influence not only whether technologies are adopted but also how they are integrated into professional practice.

The institutional perspective emphasises that organisational behaviour reflects responses to regulatory frameworks and normative expectations [27]. According to Scott [10], institutions comprise regulative, normative, and cultural–cognitive elements that provide stability and meaning to social life. In healthcare, regulations managing health information establish compliance requirements (regulative pressure) that influence technology use. Accountability mechanisms require professionals to document actions and justify decisions within established protocols. As Currie and Spyridonidis [28] demonstrated in their study of healthcare informatics, these macro-institutional logics often cascade down to the operational level, forcing clinicians to constantly reconcile top-down administrative mandates with bottom-up patient care priorities. Regardless of individual acceptance perceptions, these institutional features constitute constraints that limit the feasible scope of technology use behaviours.

Governance arrangements coordinating activities and allocating resources further shape technology application. Aman et al. [29] proved that transaction structures and decision arrangements affect how organisations manage complex operational processes. In a medical context, resource allocation decisions regarding training, technical support, and change management determine whether users possess the means to integrate new technologies into established workflows (normative support). Thus, organisational governance acts as an enabling or constraining condition for technology application.

The organisational learning process mediates the relationship between institutional conditions and sustained technology use. Kasimin et al. [30] showed that system assimilation depends on evaluation mechanisms and the organisational learning process for developing capabilities with new technologies. Technology application is not a discrete adoption event but a continuous adaptation process between technical capabilities and organisational routines. Institutional arrangements that support learning (such as the provision of training, feedback mechanisms, and management attention) facilitate the development of user competence required for sustained application.

### 2.2. Acceptance Perceptions as Behavioural Micro-Foundations

The TAM states that PU and PEU shape attitudes toward using (ATU), which in turn affect behavioural intention (BI) and subsequent actual use (AU) [31]. These relationships describe the psychological mechanisms through which evaluations of technical characteristics translate into behavioural responses.

Within the current framework, TAM constructs serve as behavioural micro-foundations, which are user-level cognitions through which institutional factors exert influence on observable behaviour. PU and PEU are not exogenous individual traits but perceptions shaped by the institutional environment. The compatibility of the technical infrastructure influences how effortless technology use appears; RC requirements affect the perception of net benefits; OS simultaneously impacts evaluations of both PU and PEU.

This positioning acknowledges the predictive validity of TAM while recognising that acceptance perceptions are modelled as endogenous variables of the institutional environment. Recent advancements in health informatics and information systems research strongly support this theoretical shift. As critically noted by Shachak et al. [32], traditional acceptance models often fall short in complex healthcare settings because they isolate user perceptions from the broader organisational context. To address this, Venkatesh et al. [14] explicitly called for the incorporation of multi-level environmental and institutional factors as critical antecedents to individual cognitions. Furthermore, Burton-Jones and Volkoff [17] demonstrated that in medical environments, a user’s evaluation of an Information Technology (IT) system is fundamentally ‘contextualised’, inextricably bound by institutional constraints, professional routines, and governance structures. Its contribution lies not in proving that PU influences ATU (which is an established relationship) but in explaining how institutional conditions shape these perceptions, thereby predicting application outcomes.

Healthcare professionals evaluate the usefulness of technology concerning work performance within institutional constraints. A system that enhances equipment tracking offers functional benefits, but if the time consumed by compliance procedures offsets the efficiency gains, this benefit may be attenuated. Similarly, ease of use depends not only on interface design but also on compatibility with existing workflows and the availability of support resources. Therefore, acceptance perceptions reflect the intersection of technological characteristics and institutional conditions. This logic is consistent with empirical evidence from Gao et al. [33], who found that in the implementation of healthcare IoT, users’ perceptions of utility and effort are profoundly reshaped by the surrounding institutional support and regulatory risk environments, confirming that individual acceptance is deeply embedded in institutional realities.

### 2.3. Hypotheses Development

#### 2.3.1. Technical Compatibility and Workflow Integration

TC refers to the degree of consistency between new technologies and existing systems, workflows, and work practices. Compatibility promotes utilisation by influencing PEU: when a system seamlessly integrates with existing infrastructure, the friction users face when incorporating new functions into their daily routines is significantly reduced [34]. In healthcare environments characterised by complex interconnected information systems (e.g., Laboratory Information System (LIS), Radiology Information System (RIS), Electronic Health Record (EHR)), compatibility determines whether new technologies complement or disrupt existing digital workflows [35].

Alauddin et al. [24] found in their study on the gig economy of digital platforms that alignment between digital tools and workers’ established labour routines is key to determining efficiency and satisfaction. This logic is equally applicable to the healthcare sector. Olson et al. [36]’s study on “Ambient AI” pointed out that technologies capable of deeply integrating into clinical dialogues without requiring additional operations most effectively reduce cognitive load. Conversely, if an IoT asset management system requires nurses to exit the EHR and log into another independent APP to locate an infusion pump, it constitutes task-switching and workflow fragmentation, greatly increasing the perceived effort.

Therefore, TC functions through knowledge transfer and skill leverage mechanisms. Professionals accumulate competencies in existing systems through long-term practice; compatible technologies allow these competencies to be transferred to new contexts, thereby reducing learning costs and enhancing PEU perceptions.

**H1.**  *TC positively influences PEU in healthcare IoT use.*

#### 2.3.2. Regulatory Compliance and Accountability Burden

A prominent feature of the healthcare environment is the presence of mandatory regulatory frameworks. In China, the PIPL classifies healthcare data as sensitive personal information, mandating strict access controls, de-identification, and operational logging. In practice, these RC requirements translate into concrete procedural steps: multi-factor authentication, mandatory data masking, and regular security audits.

While these measures are vital for privacy protection, they constitute an RC burden. In a prominent study investigating the ‘dark side’ of healthcare IT, Califf et al. [37] highlighted that excessive administrative and regulatory requirements generate significant technostress among medical staff, inadvertently detracting their cognitive resources from primary clinical duties. According to Mohaiyadin et al. [25], accountability mechanisms improve transparency but may also introduce rigidity at the operational level. In the context of healthcare IoT, when compliance procedures add operational complexity or consume time that should be devoted to clinical care, the functional benefits brought by the technology are offset by administrative costs. This explains the Privacy-Efficiency Paradox: stronger data protection is often accompanied by lower operational efficiency [38,39]. This friction is conceptualised in the healthcare digitisation literature, where boundary risks inevitably compromise ease of workflow [40].

This burden reduces not only PEU but also PU. The study by Podsakoff et al. [41] on method bias, although methodologically focused, holds a core logic applicable behaviourally: complex interfering factors distort true evaluations. In this case, RC interferes with users’ experience of the technology’s core value.

**H2a.**  *RC negatively influences PU in healthcare IoT use.*

**H2b.**  *RC negatively influences PEU in healthcare IoT use.*

#### 2.3.3. Organisational Support and Institutional Commitment

OS includes resource commitments provided for technology utilisation, such as training programmes, technical assistance, and management endorsement. Such support not only addresses capability shortages but also plays a signalling role.

Based on signalling theory, users face high uncertainty (regarding technology value, stability, and future direction) during the early stages of technology implementation. OS, as a costly signal, conveys to employees the level of importance and long-term commitment that management attaches to the technology. Martinez-Pelaez et al. [42] noted that sustainable digital transformation requires management to overcome skill barriers through cultural reshaping and resource investment.

When healthcare professionals observe that the hospital has invested in dedicated technical teams or provided protected training time, they deduce, through the signalling mechanism, that the technology is part of the organisational strategy, rather than a temporary “vanity project”. This reasoning is corroborated by Sykes [43], whose longitudinal field research proved that comprehensive support of organizational structures, such as formal training and peer advice networks, significantly alleviate transition anxiety and elevate users’ subjective evaluations of a system’s utility. This sense of trust directly elevates the assessment of the technology’s value (PU), while substantive training resources lower the learning threshold (PEU).

**H3a.**  *OS positively influences PU in healthcare IoT use.*

**H3b.**  *OS positively influences PEU in healthcare IoT use.*

#### 2.3.4. Core TAM Relationships

Consistent with existing theories, we hypothesise that institutional factors ultimately influence BI and AU through TAM’s core cognitive variables (PU, PEU, ATU) [44]. This pathway was cross-culturally validated in the meta-analysis by Veiga et al. [23].

**H4.**  *PU positively influences ATU.*

**H5.**  *PEU positively influences ATU.*

**H6.**  *ATU positively influences BI.*

**H7.**  *BI positively influences AU.*

### 2.4. Research Model and Hypothesised Relationships

**Figure 1:**
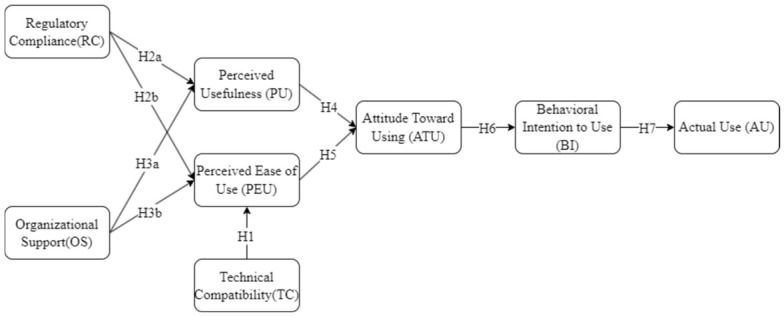
Theoretical Framework.

The theoretical framework (as shown in Figure 1) positions institutional factors (TC, RC, OS) as antecedent variables to acceptance perceptions (PU, PEU), which in turn affect AU through the mediating roles of ATU and BI. This configuration clarifies that institutional conditions constitute the cognitive foundation for technology use behaviours.

## 3. Methodology

### 3.1. Research Context and Design

This study employs a cross-sectional survey design to test the theoretical model. The research was conducted in Xinxiang City, Henan Province, China, selecting two large public hospitals representing different stages of digital maturity. Selecting China’s public hospitals as the research context is highly pertinent: first, China has advanced rapidly in healthcare digitalisation (“Internet + Healthcare”) in recent years [45]; second, with the implementation of the Data Security Law (DSL) [46] and the PIPL [20] in 2021, medical institutions face unprecedented compliance pressures, providing an ideal natural laboratory to observe the effects of regulatory burden. The selected hospitals are profiled as follows:

- **Hospital A:** A comprehensive tertiary hospital with approximately 1200 staff and 937 healthcare technical professionals. It handles about 0.87 million outpatient and emergency visits annually. Its IoT system was fully launched in 2025, equipped with a fully functional “Smart Ward” system, with core functions including intelligent infusion monitoring and bedside interactive terminals.
- **Hospital B:** A large regional medical centre with 3835 staff and approximately 3200 healthcare technical professionals. This hospital handles 2.1 million outpatient and emergency visits annually. Hospital B initiated the deployment of IoT scenarios in 2022 and simultaneously activated smart systems across its branch campuses.

The inclusion of these two hospitals permits the exploration of how different institutional configurations (a newly implemented mature system vs. an established distributed system) might yield heterogeneous effects on user perceptions and behaviours.

### 3.2. Sample and Data Collection

Data collection was conducted between September and December 2025 to ensure that respondents from both hospitals had sufficient exposure to the operational IoT systems. The inclusion criteria strictly required participants to be active frontline healthcare professionals (e.g., physicians, nurses, medical technicians, and equipment administrators) who had directly interacted with the IoT asset management systems for a minimum of three months. This criterion ensured that all respondents possessed substantial hands-on experience to accurately evaluate the system’s institutional fit and usability.

A total of 400 questionnaires were distributed via a secure online survey platform, with 200 allocated to each hospital to preserve sample balance. After excluding incomplete responses and those with patterned answers (e.g., straight-lining), 293 valid questionnaires were retained (overall effective response rate: 73.3%). Of these, 139 came from Hospital A (response rate 69.5%) and 154 from Hospital B (response rate 77.0%), yielding a near-balanced 47.4:52.6 distribution across the two institutional contexts. This symmetry supports the subsequent Multi-Group Analysis (MGA) by providing comparable group sizes (|ΔN| = 15, approximately 5% of total N), ensuring that neither hospital dominates the pooled estimates and that the MGA retains adequate statistical power in both subsamples.

To assess the potential threat of non-response bias, we followed the widely adopted extrapolation method recommended by Armstrong and Overton [47]. Independent samples *t*-tests and Chi-square tests were conducted to compare the demographic characteristics (e.g., gender, age, and professional experience) and the mean scores of core constructs between early respondents (the first 25% to complete the survey) and late respondents (the final 25%). The results indicated no statistically significant differences (*p* > 0.05) between the two groups. Consequently, non-response bias does not pose a significant threat to the validity of the findings in this study.

The detailed demographic characteristics of the respondents are presented in Table 1. Females accounted for 67.2% of the sample, which closely mirrors the broader demographic realities of the healthcare workforce in China, where nursing and allied health professions are predominantly female, thereby supporting the sample’s population representativeness. The average professional experience of the respondents was approximately 12 years, ensuring a deep understanding of both traditional clinical workflows and the newly implemented digital systems.

### 3.3. Measures

All constructs were measured using multi-item scales based on a five-point Likert scale (1 = strongly disagree to 5 = strongly agree). The scales were adapted from the established literature and modified for the healthcare IoT context (for details of the questionnaire, see Appendix A):

- **PU & PEU:** Adapted from Davis [13], referencing the validation by Veiga et al. [23] in the healthcare IoT context.
- **TC:** Drawing on Rogers’ [48] diffusion of innovation theory and Alauddin et al.’s [24] measurement of workflow integration, focusing on the fit between the system and clinical routines.
- **RC:** RC was measured with three self-developed items operationalising the procedural instantiation of the PIPL and the DSL at the level of system use. Because RC items were developed for this study rather than adapted from an existing validated instrument, content validity was established through three complementary procedures.

First, each RC item was anchored to specific legal provisions. RC1 captures the overarching obligation of lawful processing under PIPL Article 5 and the processing principles of purpose limitation and data minimisation under PIPL Articles 6 and 19, together with the sensitive personal information regime of PIPL Articles 28–29, under which health data is explicitly classified as sensitive and subject to heightened protection. RC2 captures the institutional translation of these national obligations into hospital-level operational rules, reflecting the data security management system that data processors are required to establish under DSL Article 27 and the internal compliance management obligations set out in PIPL Articles 51–52. RC3 captures the subjective procedural burden generated by the access-control, logging, masking, and audit procedures that instantiate these requirements at the point of use, and aligns conceptually with the technostress construct developed by Califf et al. [37] (a detailed item-to-provision mapping is provided in Appendix B).

Second, a comprehensibility and clarity pre-test was conducted with ten healthcare technology professionals, a purposive sample including clinical users (physicians and nurses), hospital information-technology staff, and equipment administrators, drawn from institutions other than the two target hospitals. Respondents were asked to evaluate item wording, contextual relevance, and terminological alignment with the language frontline staff use to describe compliance procedures. Feedback led to minor wording refinements of RC2 and RC3 to replace generic regulatory terminology with phrasing closer to clinical usage. We acknowledge that this procedure constitutes a pre-test of comprehensibility and face validity rather than a formal expert-panel content-validity evaluation using quantitative indices such as the Content Validity Ratio (CVR) [49] or the Content Validity Index (CVI) [50]; we flag this in Section 7 and consider replication with formal expert panel review a priority for future research.

Third, statistical evidence of dimensionality and internal consistency was obtained at both the pre-test and main-sample stages. At pre-test, Exploratory Factor Analysis (EFA) yielded a single-factor solution with all loadings exceeding 0.70 and Cronbach’s alpha above 0.80, supporting unidimensionality and reliability. In the main sample (*N* = 293), Confirmatory Factor Analysis (CFA) (reported in Section 4.1) produced standardised loadings of 0.74–0.76 (*p* < 0.001), Cronbach’s α = 0.86, Composite Reliability (CR) = 0.88, and Average Variance Extracted (AVE) = 0.58, meeting conventional thresholds for convergent validity and supporting the reflective measurement of RC.

We specify RC as a reflective construct. The three items are treated as alternative manifestations of a common latent burden and are expected to covary positively with one another (which they do, with inter-item correlations supporting unidimensionality), and dropping any single item does not alter the theoretical meaning of the construct [51,52]. This conceptualisation aligns with the dominant treatment of perceived regulatory and compliance burden in the technostress and information-systems compliance literature, where such burdens are understood as subjectively experienced states with multiple interchangeable manifestations rather than as formative composites of distinct procedural components:

- **OS:** Adapted from the Rhoades & Eisenberger [53] organisational support theory and related IT implementation studies, covering training adequacy and technical response speed.
- **ATU, BI, AU:** Standard TAM measurement items were used. It should be noted that Actual Use (AU) in this study reflects self-reported usage frequency rather than objective system log data.

To ensure the reliability and validity of the measurement tool, the questionnaire underwent a rigorous translation–back-translation procedure and was pre-tested by 10 healthcare technology professionals. During the pre-testing phase, EFA and preliminary reliability tests (Cronbach’s alpha > 0.80 for all constructs) were conducted to verify the content validity of the self-developed RC items.

### 3.4. Analytical Approach

Data analysis was conducted using Structural Equation Modelling (SEM). Given that this study is primarily based on verifying mature theories and the sample size is moderate (*N* = 293), employing Covariance-Based SEM (CB-SEM, using IBM AMOS 26.0) with Maximum Likelihood (ML) estimation is appropriate. However, regarding results reporting and quality control, we strictly adhered to the modern SEM reporting standards proposed by Hair et al. [54], particularly in reliability and validity testing and model fit evaluation. Bootstrapping with 5000 resamples was employed to rigorously test the significance of indirect (mediation) effects [55].

To address potential Common Method Bias (CMB) associated with self-reported questionnaires, this study followed the latest recommendations by Podsakoff et al. [41], adopting both procedural and statistical controls. Procedurally, psychological separation was achieved by placing independent and dependent variables in different sections of the survey, and an introductory statement explicitly guaranteed anonymity to reduce evaluation apprehension. Specifically, we conducted Harman’s single-factor test, an Unmeasured Latent Method Construct (ULMC) analysis, and assessed multicollinearity using Variance Inflation Factor (VIF) values to ensure bias remained within controllable limits.

Model fit was evaluated using standard indices: χ^2^/df < 3.0, Root Mean Square Error of Approximation (RMSEA) < 0.08, Standardised Root Mean Square Residual (SRMR) < 0.08, Comparative Fit Index (CFI) > 0.90, and Tucker–Lewis Index (TLI) > 0.90 [56].

We considered whether the nested structure of the data, respondents embedded within clinical departments, and departments within hospitals required formal multilevel modeling and concluded that CB-SEM with an explicit MGA at the hospital level is the appropriate analytical choice. At the hospital level, only two units are available (Hospital A, *n* = 139; Hospital B, *n* = 154), well below the minimum of 30 level-2 units recommended by Maas and Hox [57] for reliable multilevel SEM estimation; level-2 random effects cannot be estimated with only two clusters. At the department level, a larger number of units is available, but the stratified random sampling design, proportionally distributing respondents across clinical roles and departments, attenuates department-level clustering by construction, since within-department homogeneity is partially balanced by systematic cross-role sampling. CB-SEM with MGA is well suited to the study for the following reasons:

- The primary analytical goal is confirmatory testing of a theoretically specified model with reflective measurement, for which CB-SEM with ML estimation is appropriate [58].
- The sample size (*N* = 293) exceeds the conventional 10:1 observations-per-parameter rule for the specified model (approximately 26 free parameters).
- The MGA explicitly models the most consequential cluster, i.e., the hospital, across which institutional maturity differs, and enables substantive cross-hospital structural comparison rather than treating clustering as a nuisance to be absorbed.

We acknowledge that residual intra-department dependence may remain, and we flag this in Section 7 as a direction for future research using designs that sample larger numbers of institutions and permit three-level multilevel SEM.

## 4. Results

### 4.1. Measurement Model Evaluation

The CFA results are shown in Table 2. The standardised factor loadings of all individual items exceeded the strict 0.70 threshold (ranging from 0.74 to 0.87, *p* < 0.001), and the corresponding *t*-values indicated high statistical significance, demonstrating excellent item reliability. Internal consistency was verified by Cronbach’s α coefficients, and CR values were all above 0.80, surpassing the recommended threshold of 0.70. The AVE ranged from 0.58 to 0.65, all exceeding the 0.50 benchmark, confirming robust convergent validity. In addition, measurement errors (calculated as 1 − λ^2^) for all items were properly evaluated, and no items were removed or modified using Modification Indices (MIs), preserving the theoretical integrity of the original constructs.

### 4.2. Discriminant Validity

Discriminant validity was assessed via the Fornell–Larcker criterion (Table 3). The square roots of AVE for each construct (bold values on the diagonal) were greater than the correlation coefficients between that construct and others, demonstrating sufficient distinctiveness among the latent variables. Furthermore, per the recommendations of Hair [54] and Sarstedt et al. [59], we examined the Heterotrait–Monotrait ratio (HTMT) (Table 4); all values were below the conservative threshold of 0.85, further supporting discriminant validity.

### 4.3. Common Method Bias Testing

To verify that the observed relationships were not artificially inflated by single-source self-report, we executed a five-layer assessment combining common-method-bias controls with a direction-robustness check. First, Harman’s single-factor test revealed that the first unrotated factor accounted for only 34.6% of the total variance, well below the 50% threshold. Second, following Williams et al. [60], we introduced an ULMC, operationalised as a Common Latent Factor (CLF) connecting to all 24 observed items with equal unstandardised loadings. The CLF explained approximately 12.4% of the average item variance, substantially below levels associated with severe common-method contamination (≥25%) [61]. Third, we quantified the downstream impact on structural estimates by comparing standardised path coefficients before and after partialling out the CLF variance (Table 5).

All nine hypothesised paths retained their direction and significance. Absolute changes in β ranged from 0.02 to 0.04 (mean |Δβ| = 0.027), and relative changes ranged from 6.0% to 10.0% (mean = 7.4%), with no path exceeding either the 0.200 absolute-change or 20% relative-change thresholds recommended by Williams et al. [60] and Podsakoff et al. [41]. Fourth, VIF values ranged from 1.35 to 2.48, well below the conservative threshold of 3.3 [58], ruling out multicollinearity. Finally, to address reverse-causality concerns raised for cross-sectional designs, we compared the theoretical model against a reverse-direction specification in which cognitive perceptions were modelled as antecedents to institutional factors (Table 6). The reversed specification produced substantially poorer fit (χ^2^/df = 2.41, CFI = 0.884, RMSEA = 0.079, SRMR = 0.073; ΔCFI = −0.046 relative to the theoretical model), with CFI falling below the conventional 0.90 threshold.

While cross-sectional analysis cannot establish causation definitively, this pattern, combined with the institutional theory foundation positioning regulatory, normative, and structural pressures as antecedents to individual cognitions in professionalised contexts [10,17], provides reasonable support for the direction that we adopt. Taken together, these converging lines of evidence support the conclusion that neither CMB nor reverse causality poses a material threat to the validity of the structural estimates.

### 4.4. Structural Model and Hypothesis Testing

Before evaluating the structural model, we examined the overall goodness-of-fit of the model. The model exhibited excellent fit indices: χ^2^/df = 1.85, RMSEA = 0.062, SRMR = 0.058, CFI = 0.93, and TLI = 0.91. This indicates a strong fit between the hypothesised model and the empirical data. The path testing results of the structural model analysis are shown in Table 7. All hypothesised paths were significant at the *p* < 0.001 level, supporting all theoretical hypotheses.

The model possesses high explanatory power for the endogenous variables [58,62]: PU (R^2^ = 0.58), PEU (R^2^ = 0.60), ATU (R^2^ = 0.63), BI (R^2^ = 0.67), and AU (R^2^ = 0.60). This demonstrates that incorporating institutional factors significantly enhances the explanatory capacity for technology use behaviour.

To explicitly test the mediating role of user perceptions (the micro-foundations), bootstrapping (5000 resamples) was conducted to evaluate indirect effects (Table 8). The results confirm that institutional governance factors significantly influence downstream attitudes and self-reported use through the mediation of PU and PEU.

To ascertain whether the proposed sequential mediation chain is preferable to plausible alternatives, we estimated three competing specifications on the same measurement model (Table 9).

Model 3 augmented the baseline by adding direct paths from PU and PEU to BI, testing whether attitude mediates fully or only partially at the cognitive level. The added paths were non-significant, while the indirect paths through ATU retained their strength; fit indices improved only marginally (ΔCFI = +0.001, ΔAIC = +3.4), supporting full mediation at the cognitive level. Model 4 removed ATU entirely, letting PU and PEU predict BI directly. The fit deteriorated substantively (χ^2^/df = 2.12, CFI = 0.907, RMSEA = 0.071, SRMR = 0.067; ΔCFI = −0.023, ΔAIC = +76.2), with AIC exceeding M1 by more than 10 points, strong evidence against M4 by conventional criteria [63], and BI R^2^ decreased accordingly, confirming that ATU carries non-redundant explanatory variance that cannot be subsumed by PU and PEU alone. Model 5 extended the test of mediation to the institutional level by adding direct paths from the three institutional factors (TC, RC, OS) to BI, thereby bypassing the cognitive chain. As with Model 3, fit improved only marginally (ΔCFI = +0.002, ΔAIC = +6.9), and the added direct paths offered no substantive gain over the indirect institutional-to-cognitive-to-behavioural pathway, supporting full mediation at the institutional level. Taken together, these three comparisons converge on the theoretically grounded sequential specification (M1) as the best-fitting, most parsimonious model. More importantly, the joint support from M3 and M5 demonstrates that full mediation holds at both the cognitive and institutional levels simultaneously: institutional conditions shape behaviour through, and only through, the cognitive micro-foundations they condition, providing direct empirical validation of the theoretical architecture that positions acceptance perceptions as behavioural micro-foundations for institutional effects.

### 4.5. Multi-Group Analysis: Institutional Differences Across Hospitals

Before conducting the cross-hospital comparison, we formally evaluated measurement invariance following the standard three-step sequence [64] (results appear in Table 10).

The configural model, allowing the factor structure to vary freely across Hospital A (*n* = 139) and Hospital B (*n* = 154), achieved acceptable fit (χ^2^(426) = 812.46, χ^2^/df = 1.91, CFI = 0.924, RMSEA = 0.064, SRMR = 0.061), establishing baseline equivalence of measurement structure. Imposing metric invariance (equal factor loadings across groups) produced only a marginal decrement in fit (ΔCFI = −0.002, ΔRMSEA = 0.000), well within the thresholds of |ΔCFI| ≤ 0.010 and |ΔRMSEA| ≤ 0.015 recommended by Chen [65]. Full scalar invariance (equal item intercepts) was likewise supported (ΔCFI = −0.004, ΔRMSEA = +0.002), confirming that latent means and path coefficients are directly comparable across the two institutional settings. These results establish the validity of the subsequent structural comparisons, ensuring that any observed differences in path coefficients between Hospital A and Hospital B reflect substantive institutional heterogeneity rather than artefactual measurement differences.

To address the potential nesting effect and verify whether different institutional configurations (Hospital A: newly launched vs. Hospital B: established deployment since 2022) influence the relationships, a MGA was conducted. After establishing measurement invariance across the two groups in accordance with standard multi-group SEM reporting conventions [64], structural path comparisons revealed generally consistent directions, indicating the robustness of the core TAM chain. However, notable institutional heterogeneity was observed: the path from OS to PEU was significantly stronger in Hospital A (β = 0.42, *p* < 0.01) compared to Hospital B (β = 0.22, *p* < 0.05) (Δχ^2^ = 5.24, *p* < 0.05). This suggests that in environments with newly implemented systems (Hospital A), visible institutional commitment and training resources play a far more critical role in lowering psychological barriers than in institutions where the technology has already been routinised.

The selective heterogeneity observed in the MGA, with OS → PEU differing markedly across hospitals (β_A = 0.42 vs. β_B = 0.22, Δχ^2^ = 5.24, *p* < 0.05) while RC → PU, RC → PEU, and TC → PEU remain statistically indistinguishable, warrants a theoretically principled reading rather than a post hoc interpretation. Three explanatory channels are consistent with the data. First, the RC pathway reflects a macro-institutional force exerted uniformly through the PIPL and DSL; both hospitals operate under identical legal obligations and face equivalent procedural instantiations, leaving no structural basis for cross-hospital variation in this mechanism. Second, TC operates through a cognitive mechanism, namely the reduction in task-switching and workflow fragmentation, that is relatively invariant across implementation maturity stages: the cognitive cost of switching applications is the same whether the IoT system was launched last year or three years ago. Third, OS carries a signalling function whose salience is intrinsically time-varying [48,66]: during the early transition window characterising Hospital A, support mechanisms reduce uncertainty and signal institutional commitment, amplifying their perceived effect; once the technology is routinised, as in Hospital B, this signalling channel attenuates because the technology is already legitimised and support reverts to its instrumental role. We ruled out two alternative explanations: statistical power is unlikely to account for the null differences on other paths (*n*(A) = 139, *n*(B) = 154 are both adequate for SEM path comparisons), and full scalar measurement invariance was established for all constructs (Table 10), precluding measurement-level artefacts.

## 5. Discussion

### 5.1. Institutional Conditions as Antecedent Determinants of Perceptions

The research findings suggest that in regulated healthcare environments, technology application relies on institutional configurations that shape user perceptions. Although PU and PEU predict ATU and BI as always, confirming the role of cognitions as the micro-foundations of behaviour, it is more important to note that these cognitions themselves are highly dependent on institutional factors. This antecedent dependency constitutes the core explanatory contribution of this study: individual acceptance is insufficient to explain utilisation; institutional conditions determine whether acceptance can be transformed into sustained practice. The MGA results further support this by demonstrating that variations in institutional maturity alter the strength of support mechanisms.

The high variance explanation rates of PU (58%) and PEU (60%) show that healthcare workers’ evaluation of technology is not based on abstract functional parameters but on actual usage conditions, namely infrastructure compatibility, compliance burden, and the availability of support. This finding echoes the classic assertion by Orlikowski & Barley [67] that the utility of IT is not embedded within the technology itself but constructed in practice by the institutional structures in which it is embedded.

### 5.2. Technical Compatibility and Workflow Integration Mechanisms

The significant positive association between TC and PEU (β = 0.40) reveals the critical role of workflow integration. Medical work is inherently highly intense and fragmented and carries a high cognitive load. Carayon et al. [68] pointed out in their research that any new tool breaking the equilibrium of existing clinical work systems would trigger intense cognitive resistance and operational workarounds from healthcare professionals. If an IoT asset management system requires staff to switch frequently among EHR, LIS, and asset APPs, this “cognitive fragmentation” will drastically lower ease-of-use perceptions.

Conversely, high compatibility means that the technology can merge into existing workflows. Research by Olson et al. [36] and Alauddin et al. [24] both indicate that technologies capable of automatically embedding into clinical dialogues without extra clicks best reduce administrative burdens. In this study, IoT devices seamlessly integrated with the hospital’s existing information systems allow healthcare professionals to utilise their existing operational skills and cognitive schemas, thus gaining new functions without adding extra learning costs. This suggests that for healthcare IT procurement, interoperability is not just a technical metric but the core determinant of user experience.

For healthcare institutions, this implies that technology selection should prioritise integration with existing infrastructure over feature richness. A system that is technologically superior but disrupts established workflows may actually have a lower application rate than a moderately featured system that integrates seamlessly with existing practices.

### 5.3. Regulatory Compliance and the Attenuation of Technology Benefits

The negative correlations between RC and both PU (β = −0.25) and PEU (β = −0.20) reveal how accountability structures attenuate technology benefits in regulated professional environments. This finding is of significant importance for understanding technology application in healthcare.

Healthcare professionals operate within an accountability framework demanding documentation, authorisation, and audit compliance. Mohaiyadin et al. [25] proved that accountability mechanisms fundamentally shape how organisations implement technological solutions. In the context of IoT, compliance requirements impose procedural steps, such as access authentication, usage logging, and data processing protocols, which consume time and attention. These requirements do not eliminate the benefits of technology but attenuate them: net usefulness equals gross functional benefit minus compliance costs.

The negative impact on PU indicates that medical professionals assess utility based on net benefits rather than total benefits. A system saving time through efficient equipment location might still be viewed as less useful if compliance procedures consume the time saved. Given the institutional accountability context, this calculation is rational: professionals must comply with regulations whether they use the technology or not, hence compliance costs represent a practical deduction of technological value.

The negative impact on PEU suggests that compliance requirements increase the complexity of the usage process itself. Beyond functional system operations, users must navigate compliance procedures that add cognitive and procedural demands. Therefore, the accountability environment shapes PEU independently of interface design or technical complexity.

These findings suggest a tension between regulatory objectives and technology application. Regulations governing healthcare data protection play a crucial role in safeguarding patient privacy and ensuring service quality. However, the costs imposed by compliance mechanisms may hinder the adoption of beneficial technologies. Resolving this tension requires approaches that achieve regulatory goals while minimising procedural burdens, potentially through a “compliance-by-design” approach that embeds regulatory requirements within system architecture rather than overlaying them as extra procedures.

### 5.4. Organisational Support and Institutional Commitment Signals

The positive impacts of OS on PU (β = 0.35) and PEU (β = 0.30) demonstrate that institutional commitment shapes user perceptions through multiple mechanisms.

Firstly, providing support demonstrates that technology applications are valued within the organisation. When management endorses technology adoption and allocates resources for implementation, professionals perceive an alignment between technology use and organisational goals. Aman et al. [69] showed that a climate of trust influences behavioural outcomes through perceived organisational commitment. In a technological context, providing support creates a climate of trust where users believe the technology investment serves institutional purposes they agree with, thereby enhancing perceived usefulness through goal alignment.

Secondly, OS promotes capability development through resource provision. Training programmes offer structured skill acquisition opportunities; the availability of technical assistance alleviates anxieties regarding implementation difficulties. Kasimin et al. [30] proved that organisational learning relies on evaluation and support mechanisms that facilitate system assimilation. The provision of support creates conditions for learning processes through which users develop proficiency, directly enhancing PEU.

Thirdly, support mechanisms enable potential technological benefits, which might otherwise remain latent, to be realised. Technologies provide functional capabilities, but users must develop skills to harness them. Without training and assistance, users might only operate systems at minimum functional levels, perceiving limited usefulness. Support provision enables deeper engagement, thereby unveiling fuller potential benefits.

The dual impact on PU and PEU suggests that OS operates as a comprehensive facilitating condition, rather than just addressing specific barriers. Organisations investing in support create general conditions conducive to technology application; those lacking adequate support leave users devoid of the institutional resources necessary for successful integration.

Building on these general mechanisms, more granular reading emerges from the MGA. The finding that from OS to PEU is substantially stronger in Hospital A, where the system was newly launched in 2025, than in Hospital B, where deployment dates back to 2022 (β_A = 0.42 vs. β_B = 0.22; Δχ^2^ = 5.24, *p* < 0.05), warrants a theoretical reading that extends beyond the skill-acquisition framing typical of the training literature. During the early transition phase, healthcare professionals confront substantive uncertainty along three dimensions: whether the technology will persist, whether their personal investment in learning it will be rewarded, and whether management endorsement is authentic rather than ceremonial. Under these conditions, training programmes and dedicated technical assistance function less as vehicles for competence transfer and more as costly signals of organisational commitment in the sense developed by Spence [66].

The signalling interpretation requires three conditions to hold, each of which is observable in hospital practice. First, the signal must be costly to the sender: protected training hours scheduled during clinical time (rather than in addition to it), dedicated technical teams staffed with experienced engineers, and executive sponsorship that visibly consumes senior management attention all satisfy this condition, because they foreclose alternative uses of scarce institutional resources. Second, the signal must be observable to the receiver: frontline staff must see that their unit managers are releasing them for training, that technical support is responsive rather than symbolic, and that senior administrators attend key deployment milestones rather than delegating attendance. Third, the signal must be aligned with the sender’s demonstrated interest in the receiver’s success: recurring rather than one-off training, peer champion networks that outlive the launch phase, and feedback mechanisms that visibly inform subsequent iterations of the system all reinforce credibility. Where any of these three conditions fail, OS can be present in name but generate no signalling value, leaving users in the state of uncertainty that the transition phase would otherwise resolve.

Once the technology is routinised, as in Hospital B, where deployment dates back three years and has extended across branch campuses, this signalling channel attenuates. The system is already institutionalised, uncertainty about its persistence is resolved, and support mechanisms revert to their instrumental function of competence maintenance. This attenuation is not evidence that support ceases to matter: the β = 0.22 coefficient in Hospital B remains statistically significant. Rather, the theoretical function of support shifts from trust-building (during transition) to capability-sustaining (in routinised settings), and its marginal effect on PEU correspondingly diminishes.

Two managerial implications follow. First, during the first twelve to twenty-four months after deployment, the transition window, OS should be deliberately designed as a trust-building apparatus, with visible executive sponsorship, training hours protected during clinical time, peer champion networks, and sustained rather than pulsed technical assistance. Generic “train-and-release” patterns risk discharging the formal requirement of support without generating the signalling value that our findings indicate is load bearing in this phase. Second, in routinised settings, support allocation can legitimately transition toward helpdesk efficiency and periodic refresher training, but hospitals should guard against premature reduction in support during the transition window, when the signalling function remains load bearing. Failure to invest in visible institutional commitment during the transition phase risks cascading into perception deterioration that is difficult to reverse once first impressions have crystallised.

### 5.5. The Mediating Structure of Institutional Effects

The model’s mediating structure, where institutional factors influence application through acceptance perceptions, provides insights into how institutional conditions shape behaviour. Institutional factors do not determine application directly; rather, they shape the cognitive evaluations professionals use to assess technology. This mediating role implies that institutional conditions exert influence by altering users’ perceptions of technological features, rather than by directly restricting behaviour.

The practical implication is that institutional interventions can enhance technology application by transforming user perceptions. Improving TC does not merely reduce implementation barriers; it alters users’ perceptions of ease of use. Providing OS does not merely make application possible; it alters users’ evaluations of usefulness and effort requirements. Therefore, institutional factors influence application through cognitive mechanisms rather than purely structural ones.

This cognitive mediation suggests that institutional effects may be more malleable than structural constraints imply. Organisations can shape technology perceptions through compatibility investments, compliance workflow simplification, and support provision, thereby influencing application outcomes without modifying the technical features themselves.

### 5.6. Professional Responsibility and Healthcare-Specific Application Dynamics

These findings must be interpreted within the unique context of healthcare professional responsibilities. Healthcare workers operate under dual accountability structures: an organisational hierarchy that allocates tasks and evaluates performance, and professional norms that define standards of care and ethical obligations. Technology application occurs at the intersection of these accountability systems.

The negative RC effects likely reflect a tension between efficiency-oriented technological benefits and accountability-oriented compliance requirements. Healthcare professionals are socialised to prioritise patient safety and regulatory adherence; when compliance procedures appear to conflict with operational efficiency, they rationally prioritise compliance over efficiency. Therefore, the attenuation of PU does not represent a failure in technology design but a rational response to accountability priorities.

This interpretation suggests that technology implementation in healthcare must be positioned within accountability frameworks, rather than in opposition to them. Technologies that enhance compliance via automated documentation, audit trail generation, or integrated regulatory checks might achieve higher application rates than those competing with compliance for professional attention. The challenge for technology designers is to align operational efficiency gains with accountability enhancements, rather than placing them in tension.

OS effects similarly reflect healthcare-specific dynamics. Healthcare institutions operate via complex coordination mechanisms, integrating diverse professional groups with different capabilities and practice patterns. Alauddin et al. [24] demonstrated that digital platform governance shapes worker behaviour through structural arrangements coordinating activities across distributed contexts. In healthcare, OS mechanisms play a similar role, coordinating technology integration across professional boundaries and departmental structures.

Therefore, effective support requires attention to the coordination challenges unique to healthcare environments. Training programmes must accommodate diverse professional backgrounds; technical support must be accessible across shift patterns and unit locations; management endorsement must bridge professional silos. Generic support approaches may prove inadequate; healthcare-specific support strategies tailored to coordinate the complexities of multi-professional environments are required.

### 5.7. Implications for Healthcare Sustainability

The findings of this study provide critical implications for modern healthcare management and clinical operational efficiency. Efficient IoT asset management directly enhances the utilisation rate of medical resources and reduces equipment idling, thereby supporting a more resilient health system [70]. For instance, effectively integrating IoT systems with existing workflows can significantly reduce administrative redundancy, which not only optimises the lifecycle management of expensive medical equipment but also substantially alleviates the severe workloads of frontline medical staff.

Technology governance in healthcare has traditionally focused on selection and procurement: evaluating technology options, negotiating contracts, and managing implementation projects. The research findings suggest that ongoing governance—the institutional arrangements within which application occurs—may be equally important. Technologies do not fail at implementation; their success or failure depends on sustained application relying on continuous institutional support.

This perspective implies that technology governance should extend beyond initial implementation to the continuous assessment of institutional conditions. Are existing systems compatible with newly added technologies? Do compliance requirements impose excessive burdens? Is the OS adequate and continuous? Regular evaluations of these institutional conditions can identify application barriers before they lead to implementation failure.

The significant variance explained by institutional factors (58–67% across constructs) demonstrates that governance interventions can meaningfully affect application outcomes. This explanatory power indicates that technology application is not primarily determined by fixed technical characteristics or immutable user preferences but by malleable institutional conditions susceptible to management interventions. Therefore, optimising institutional governance is not merely an administrative issue but a critical pathway for achieving healthcare sustainability.

## 6. Contributions and Implications

### 6.1. Theoretical Contributions

This study explains the institutional conditions under which technology application occurs in regulated professional environments. The mainstream approach to technology adoption research provides limited insights into why application succeeds or fails in specific institutional contexts. By positioning acceptance perceptions as mediating mechanisms translating institutional factors into behaviour, this study explains how institutional configurations shape application outcomes.

The findings indicate that individual acceptance is necessary but not sufficient for sustained technology application. If institutional conditions, such as regulatory burden, insufficient support, and infrastructural incompatibility, undermine the relationship between acceptance and behaviour, healthcare professionals who perceive the technology as useful and easy to use may still underutilise existing systems. Conversely, institutional arrangements that enhance perceptions and facilitate integration can promote application even when initial acceptance is moderate.

This institutional governance perspective extends technology acceptance research, specifying the conditions under which core TAM relationships operate. By shifting the focus from general IT adoption to deeply embedded organisational routines, this study answers the research of Hong et al. [71] call to develop context-specific theories in information systems, proving that generic models must be bounded by specific institutional constraints

The scope of this theoretical contribution warrants explicit statement. The institutional governance perspective developed here is intended to apply to regulated healthcare IoT broadly; however, empirical support for the specific coefficient magnitudes reported (e.g., TC → PEU β = 0.40, RC → PU β = −0.25) is anchored in the asset-management use case. Generalisation of the theoretical architecture to other healthcare IoT domains is plausible but requires replication; the coefficient magnitudes themselves should be read as context-specific estimates rather than universal constants.

### 6.2. Methodological Value

This study demonstrates the value of integrating acceptance models with institutional perspectives in regulated professional contexts. The mediating model structure enables the examination of how institutional factors operate through cognitive mechanisms, providing richer explanations than direct-effect approaches. This integration offers a template for researching technology application in other regulated professional environments, such as legal services, financial institutions, and public administration, where institutional factors similarly shape the conditions of use.

### 6.3. Practical Implications

#### 6.3.1. Technical Infrastructure

It is important to prioritise compatibility with existing systems during technology selection. Investing in integration middleware or interface adaptation may enhance application more effectively than investing in advanced features that disrupt established workflows. Compatibility assessments should precede procurement decisions and inform technical specifications.

Translating the β = 0.40 coefficient for TC into procurement practice requires moving from implicit heuristics to explicit prioritisation rules. In current hospital procurement, committees often default to feature-count comparisons, more sensors, broader analytics suites, and richer dashboards, because features are easier to score than integration depth. Our findings indicate that this heuristic is structurally misaligned with the determinants of sustained use in regulated environments. We propose a procurement scorecard that weights interoperability above functional breadth, organised in three tiers.

The first tier comprises gating requirements that candidate systems must satisfy before functional features are even scored. These include the following:

- Native conformance with Health Level Seven (HL7) Fast Healthcare Interoperability Resources (FHIR) R4 for device data exchange, and with Integrating the Healthcare Enterprise (IHE) Patient Care Device (PCD) profiles for medical-equipment integration.
- Demonstrated bi-directional integration with the incumbent EHR and clinical data repository, verified in a time-bounded pilot within an active clinical unit rather than through vendor self-assertion or sandbox demonstration.
- Support for the hospital’s existing identity and access management layer, including Single Sign-On (SSO).
- The availability of open application programming interface (API) permitting the hospital to add, substitute, or decommission components without vendor lock-in.

Systems that fail any of these gates are excluded from further evaluation regardless of functional performance, because workflow integration problems revealed after procurement are substantially more costly to remediate than foregone features.

The second tier evaluates workflow fit using criteria derived directly from the mechanism by which TC affects PEU, the reduction in cognitive fragmentation and task-switching. Relevant criteria include the number of context switches required for common tasks, the degree of visual and interaction consistency with the incumbent clinical interface, and the alignment of system-generated alerts with established escalation protocols. Olson et al. [36] demonstrate that ambient, context-aware tools most effectively reduce clinician cognitive load; procurement evaluation should therefore measure how closely the candidate system approximates this ambient quality within the hospital’s existing operational environment, rather than simply cataloguing the features it adds.

The third tier, engaged only after the first two are satisfied, evaluates functional breadth, sensor coverage, analytics capabilities, and reporting depth. At this stage, richness serves as a differentiator among otherwise comparable candidates, not as a substitute for interoperability. This sequencing mirrors the priority structure suggested by the empirical findings: absent compatibility, feature richness cannot be realised in sustained use.

Finally, pilot testing before scale-up should occur within an active clinical unit during routine operations rather than within a controlled sandbox because workflow fragmentation emerges only when the system meets the real rhythm of clinical work. A structured pilot of fourteen to twenty-eight days, with feedback solicited from at least three role categories (physician, nurse, technical administrator) and monitoring of concrete workflow metrics such as context-switching frequency, authentication events per shift, and task-completion time, provides a far stronger evidentiary base for procurement decisions than vendor demonstrations conducted outside of live clinical pressure. This procurement discipline translates the theoretical finding, that interoperability is not merely a technical metric but the core behavioural determinant of sustained utilisation, into an actionable institutional practice.

#### 6.3.2. Regulatory Compliance

Seek compliance approaches that minimise procedural burdens while achieving protection goals. A “compliance-by-design” architecture embedding regulatory requirements into system functions reduces compliance costs that attenuate PU. Collaboration between technology implementers and RC officers during system configuration can identify opportunities to streamline compliance workflows.

The negative coefficients for RC (RC → PU: β = −0.25; RC → PEU: β = −0.20) indicate that the behavioural cost of compliance arises principally at the user layer, where clinicians shoulder procedural steps that could in principle be absorbed elsewhere in the system. A compliance-by-design architecture operationalises this insight by shifting regulatory obligations from the clinical interface to the data and infrastructure layers. In the Chinese regulatory context shaped by the PIPL and the DSL, this shift is particularly consequential because medical data is classified as sensitive personal information subject to heightened protection. We outline four architectural principles through which this shift can be operationalised in healthcare IoT asset management systems.

First, at the identity and access layer, federated SSO tied to the hospital’s existing EHR identity service, combined with role-based or attribute-based access control (Role-Based Access Control (RBAC)/Attribute-Based Access Control (ABAC)), allows clinicians to authenticate once rather than repeatedly for each IoT-enabled device or sub-system. Access permissions are inherited from the clinical role (e.g., attending physician, registered nurse, equipment administrator) and the operational context (ward, shift, equipment class), removing from the user the cognitive task of reasoning about permission scopes.

Second, at the data layer, pseudonymisation and minimisation are enforced at ingestion. Direct patient identifiers are replaced with purpose-specific pseudonyms at the point where IoT device telemetry enters the hospital data plane; full re-identification is restricted to narrowly defined administrative workflows and logged automatically. Under this design, the PIPL obligations governing data minimisation and purpose limitation are discharged by the pipeline rather than by a clinical user deciding, in the moment, which fields to redact.

Third, at the governance layer, immutable event-driven audit trails replace manual documentation. Every access, query, and data flow is recorded in append-only storage with cryptographic integrity guarantees, generating the audit evidence required by DSL Article 29 and PIPL Article 55 without asking staff to produce secondary compliance records. Compliance officers gain the continuous monitoring they need, while clinicians are relieved of the overhead documentation that attenuates PU.

Fourth, consent-aware API gateways enforce purpose limitation programmatically. When IoT-collected data is requested for a secondary use, the gateway checks the original processing purpose registered for that data and either permits, denies, or re-anonymises the flow according to policy; no human intermediary is required to interpret permissible use in real time. This transforms consent management from a recurring user decision into a systemic guarantee.

Implementing this architecture requires early collaboration between information-technology vendors, hospital compliance officers, and clinical champions during system specification, because retrofitting compliance controls after procurement typically reintroduces the very procedural burden the approach aims to eliminate. The practical implication of our findings on RC is therefore not merely legal but behavioural: absent architectural absorption of regulatory obligations, the benefits of IoT investment are systematically eroded at the point of use, and the Privacy–Efficiency Paradox persists as an operational reality rather than a design problem awaiting solution.

#### 6.3.3. Organisational Support

Investment in comprehensive support programmes encompassing training, technical assistance, and visible management endorsement is necessary. The provision of support shapes perceptions through capability development and institutional commitment signals. Support should continue beyond initial implementation; continuous training and readily available technical assistance maintain the institutional conditions that facilitate application.

#### 6.3.4. Governance Integration

Technology implementation should be understood as a continuous governance challenge rather than a discrete project. Regular assessments of institutional conditions—compatibility with evolving infrastructure, the adequacy of support provision, and the burden of compliance requirements—can identify emerging barriers before they derail application.

## 7. Limitations and Future Research

Although this study provides profound insights, limitations exist that necessitate careful interpretation of the findings.

First, the cross-sectional design restricts the capability for causal inference. Consequently, the possibility of reverse causality—for example, higher usage leading to a greater awareness of regulatory burdens—cannot be fully excluded, although the reverse-direction model test reported in Section 4.3 provides supporting evidence for the theoretically posited causal direction. Endogeneity concerns driven by unobserved heterogeneity (e.g., individual digital literacy or department-specific workload) also persist. Future longitudinal studies could track this dynamic process. Beyond restricting causal inference, the cross-sectional design limits our ability to observe how institutional conditions and user perceptions co-evolve across the implementation lifecycle. Our three-month use criterion ensures that respondents are past the initial familiarisation phase but does not capture whether the observed relationships attenuate, intensify, or reverse over a longer horizon. Future research should combine periodic survey waves with system-generated log data to decompose sustained utilisation into its behavioural components and triangulate against psychological self-reports.

Second, AU in this study was operationalised through self-reported survey items rather than objective system log data. While rigorous statistical remedies (e.g., CLF) were deployed to mitigate CMB, self-reported usage remains vulnerable to social desirability and consistency motifs. Future research must incorporate objective backend log data to construct a multi-source dataset, effectively bridging the gap between psychological intention and verifiable behavioural traces.

Third, the single-country context (China) may limit the generalisability of the results. China’s PIPL regulations and the administrative governance structures of its public hospitals are distinctive. Future comparative research could validate the applicability of this model in countries with different regulatory environments to assess the boundary conditions of institutional factors.

Fourth, while the theoretical narrative suggests that institutional factors are “necessary” conditions for sustained usage, SEM inherently tests for sufficiency and associations. Future studies could employ Necessary Condition Analysis (NCA) to definitively establish the bottleneck thresholds of these institutional constraints.

Fifth, while we established content validity for the self-developed RC scale through legal-textual anchoring to PIPL and DSL provisions, a pre-test with ten healthcare technology professionals, and confirmatory factor analytic evidence, we did not conduct a formal expert-panel content validation using quantitative indices such as Lawshe’s [49] CVR or the CVI [50]. The replication of the RC scale through formal panel-based content validation and its cross-validation in jurisdictions operating under distinct privacy regimes (e.g., GDPR in the European Union, Health Insurance Portability and Accountability Act (HIPAA) in the United States) remain priorities for future work.

Sixth, our empirical focus on hospital IoT-based asset management systems means that the observed relationships, while theoretically applicable to healthcare IoT more broadly, have been tested in one specific class of use cases. Asset management systems have distinctive characteristics, relatively mature deployment histories, well-defined workflow integration points with existing hospital information systems, and moderate data-sensitivity compared with direct patient monitoring, which may modulate the strength or nature of the institutional-governance-to-use pathway. The replication of the framework in other healthcare IoT categories, such as continuous physiological monitoring systems, tele-intensive-care platforms, and patient-worn IoT devices subject to more direct clinical-safety regulation, is a priority for future research. Designs sampling larger numbers of institutions would also permit formal three-level multilevel SEM to address residual intra-department dependence that the current analysis, with only two hospitals, cannot model directly.

Lastly, future research should examine how institutional conditions evolve during technology implementation and how initial conditions shape application trajectories. Comparative studies across regulatory environments will evaluate the applicability boundary conditions of the observed relationships. Investigations into “compliance-by-design” approaches will offer practical guidance for reconciling regulatory goals with application enhancement.

## 8. Conclusions

This study demonstrates that in regulated healthcare environments, technology application is fundamentally an issue of institutional governance. While individual acceptance remains essential, sustained application is strongly associated with the degree of alignment between technological capabilities and institutional conditions. Specifically, regulatory burdens constitute a significant bottleneck that reduces the perceived value of IoT systems. To achieve the goals of resilient and sustainable healthcare systems, medical institutions must move beyond simple procurement models. Instead, they should invest in more effective governance configurations characterised by high interoperability, automated compliance, and robust institutional support, thereby transforming digital potential into clinical reality.

Overall, this study emphasises that sustainable digital transformation in healthcare is fundamentally a governance challenge rather than merely a technological one. Aligning technological capabilities with institutional conditions through interoperability, streamlined compliance, and sustained OS represents a critical pathway for ensuring that digital health infrastructures effectively contribute to sustainable healthcare systems.

## Figures and Tables

**Table 1:** Demographic characteristics of the respondents (*N* = 293).

Demographic Profile	Category	Frequency (n)	Percentage (%)
Gender	Male	96	32.8
	Female	197	67.2
Age (years)	20–30	82	28.0
	31–40	108	36.9
	41–50	68	23.2
	>51	35	11.9
Profession	Physician	83	28.3
	Nurse	176	60.1
	Other (Tech/Admin)	34	11.6
Professional Experience (years)	<5	60	20.5
	5–10	100	34.1
	11–20	89	30.4
	>20	44	15.0

**Table 2:** Confirmatory Factor Analysis Results.

Construct	Items	Standardised Loading (λ)	Measurement Error (1 − λ^2^)	*t*-Value (*p* < 0.001)	Cronbach’s α	AVE	CR
PU	PU1	0.74	0.45	13.52	0.87	0.58	0.88
	PU2	0.77	0.41	14.28			
	PU3	0.80	0.36	15.10			
PEU	PEU1	0.78	0.39	14.45	0.88	0.60	0.89
	PEU2	0.80	0.36	15.02			
	PEU3	0.82	0.33	15.71			
ATU	ATU1	0.77	0.41	14.20	0.89	0.62	0.90
	ATU2	0.80	0.36	15.15			
	ATU3	0.84	0.29	16.33			
BI	BI1	0.82	0.33	15.68	0.90	0.63	0.91
	BI2	0.83	0.31	16.05			
	BI3	0.85	0.28	16.82			
AU	AU1	0.84	0.29	16.20	0.91	0.65	0.92
	AU2	0.85	0.28	16.65			
	AU3	0.87	0.24	17.40			
TC	TC1	0.78	0.39	14.30	0.89	0.61	0.90
	TC2	0.79	0.38	14.65			
	TC3	0.80	0.36	14.98			
RC	RC1	0.74	0.45	13.40	0.86	0.58	0.88
	RC2	0.75	0.44	13.65			
	RC3	0.76	0.42	13.92			
OS	OS1	0.80	0.36	15.05	0.89	0.62	0.90
	OS2	0.81	0.34	15.40			
	OS3	0.83	0.31	15.95			

Note: Measurement error is calculated as 1 minus the square of the standardised loading.

**Table 3:** Discriminant Validity Matrix.

	PU	PEU	ATU	BI	AU	TC	RC	OS
PU	**0.76**							
PEU	0.60	**0.78**						
ATU	0.63	0.57	**0.79**					
BI	0.58	0.52	0.67	**0.79**				
AU	0.55	0.50	0.62	0.70	**0.81**			
TC	0.48	0.63	0.51	0.49	0.45	**0.78**		
RC	−0.45	−0.40	−0.37	−0.35	−0.32	−0.30	**0.76**	
OS	0.50	0.55	0.48	0.46	0.44	0.62	−0.42	**0.79**

Note: Diagonal elements in bold are the square roots of AVE; off-diagonal elements are correlation coefficients.

**Table 4:** Heterotrait–Monotrait Ratio Matrix.

	PU	PEU	ATU	BI	AU	TC	RC	OS
PU	-							
PEU	0.68	-						
ATU	0.71	0.64	-					
BI	0.65	0.58	0.74	-				
AU	0.62	0.56	0.69	0.77	-			
TC	0.54	0.71	0.57	0.55	0.50	-		
RC	0.51	0.45	0.42	0.39	0.36	0.34	-	
OS	0.56	0.62	0.54	0.51	0.49	0.70	0.48	-

**Table 5:** Pre/Post-CLF Path Coefficient Comparison.

Hypothesis	Path	β (Pre-CLF)	β (Post-CLF)	|Δβ|	% Change
H1	TC → PEU	0.40	0.37	0.03	7.5%
H2a	RC → PU	−0.25	−0.23	0.02	8.0%
H2b	RC → PEU	−0.20	−0.18	0.02	10.0%
H3a	OS → PU	0.35	0.32	0.03	8.6%
H3b	OS → PEU	0.30	0.28	0.02	6.7%
H4	PU → ATU	0.45	0.42	0.03	6.7%
H5	PEU → ATU	0.30	0.28	0.02	6.7%
H6	ATU → BI	0.50	0.47	0.03	6.0%
H7	BI → AU	0.60	0.56	0.04	6.7%
**Mean**	—	—	—	**0.027**	**7.4%**
**Maximum**	RC → PEU	—	—	**0.04**	**10.0%**

Note: Bold values in the bottom two rows (Mean and Maximum) indicate summary statistics aggregating the nine path-specific changes reported above.

**Table 6:** Reverse-Direction Robustness Check.

Model	χ^2^/df	CFI	RMSEA	SRMR	ΔCFI vs. M1
M1 Theoretical direction (institutional factors → cognitions → behaviour)	1.85	0.930	0.062	0.058	—
M2 Reversed direction (cognitions → institutional factors)	2.41	0.884	0.079	0.073	−0.046

**Table 7:** Structural Equation Modelling Path Coefficients.

Hypothesis	Path	β	SE	*t*-Value (*p* < 0.001)	Result
H1	TC → PEU	0.40	0.06	6.32	Supported
H2a	RC → PU	−0.25	0.05	−5.26	Supported
H2b	RC → PEU	−0.20	0.03	−6.32	Supported
H3a	OS → PU	0.35	0.06	5.53	Supported
H3b	OS → PEU	0.30	0.05	6.32	Supported
H4	PU → ATU	0.45	0.06	7.11	Supported
H5	PEU → ATU	0.30	0.05	6.32	Supported
H6	ATU → BI	0.50	0.08	6.32	Supported
H7	BI → AU	0.60	0.09	6.32	Supported

**Table 8:** Bootstrap Indirect Effects for Mediation Testing.

Path	Specific Indirect Effect (β)	95% CI Lower	95% CI Upper	Result
TC → PEU → ATU	0.120	0.085	0.162	Significant
RC → PU → ATU	−0.113	−0.158	−0.076	Significant
RC → PEU → ATU	−0.060	−0.092	−0.035	Significant
OS → PU → ATU	0.158	0.112	0.210	Significant
OS → PEU → ATU	0.090	0.058	0.130	Significant

Note: All indirect effects are significant at *p* < 0.001. Moreover, 95% Confidence Intervals (CIs) were generated using 5000 bootstrap resamples. Significance is established if the CI does not contain zero.

**Table 9:** Comparison of Alternative Model Specifications.

Model	Specification	χ^2^/df	CFI	RMSEA	SRMR	AIC	ΔAIC vs. M1
M1	Hypothesised model (PU/PEU → ATU → BI → AU)	1.85	0.930	0.062	0.058	1012.4	baseline
M3	Add direct paths PU → BI and PEU → BI (tests full vs. partial mediation at cognitive level)	1.84	0.931	0.061	0.057	1015.8	+3.4
M4	ATU removed; PU and PEU predict BI directly	2.12	0.907	0.071	0.067	1088.6	+76.2
M5	Add direct paths from TC, RC, OS to BI (tests full vs. partial mediation at institutional level)	1.83	0.932	0.061	0.057	1019.3	+6.9

Note: AIC = Akaike Information Criterion.

**Table 10:** Measurement Invariance.

Model	χ^2^	df	χ^2^/df	CFI	RMSEA	SRMR	ΔCFI	ΔRMSEA
Configural	812.46	426	1.91	0.924	0.064	0.061	—	—
Metric (loadings equal)	829.37	442	1.88	0.922	0.064	0.063	−0.002	0.000
Scalar (intercepts equal)	861.58	458	1.88	0.918	0.066	0.065	−0.004	+0.002

## Data Availability

The data presented in this study are available on request from the corresponding author due to the internal privacy policies and data protection regulations of the participating hospitals, which strictly prohibit the public sharing of employee survey data without prior administrative authorisation.

## Abbreviations

The following abbreviations are used in this manuscript:

ABAC	Attribute-Based Access Control
AIC	Akaike Information Criterion
API	Application Programming Interface
ATU	Attitude Toward Using
AU	Actual Use
AVE	Average Variance Extracted
BI	Behavioural Intention
CB-SEM	Covariance-Based Structural Equation Modelling
CFA	Confirmatory Factor Analysis
CFI	Comparative Fit Index
CI	Confidence Interval
CLF	Common Latent Factor
CMB	Common Method Bias
CR	Composite Reliability
CVI	Content Validity Index
CVR	Content Validity Ratio
DSL	Data Security Law
EFA	Exploratory Factor Analysis
EHR	Electronic Health Record
FHIR	Fast Healthcare Interoperability Resources
GDPR	General Data Protection Regulation
HIPAA	Health Insurance Portability and Accountability Act
HL7	Health Level Seven
HTMT	Heterotrait–Monotrait Ratio
IHE	Integrating the Healthcare Enterprise
IoT	Internet of Things
IT	Information Technology
LIS	Laboratory Information System
MGA	Multi-Group Analysis
MI	Modification Indices
ML	Maximum Likelihood
NCA	Necessary Condition Analysis
OS	Organisational Support
PCD	Patient Care Device
PEU	Perceived Ease of Use
PIPL	Personal Information Protection Law
PU	Perceived Usefulness
RBAC	Role-Based Access Control
RC	Regulatory Compliance
RIS	Radiology Information System
RMSEA	Root Mean Square Error of Approximation
SEM	Structural Equation Modelling
SRMR	Standardised Root Mean Square Residual
SSO	Single Sign-On
TAM	Technology Acceptance Model
TC	Technical Compatibility
TLI	Tucker–Lewis Index
ULMC	Unmeasured Latent Method Construct
UTAUT	Unified Theory of Acceptance and Use of Technology
VIF	Variance Inflation Factor

## Appendix A. Survey Questionnaire

Part 1: Demographics

Gender: □ Male □ Female

Age: □ 20–30 □ 31–40 □ 41–50 □ >51

Profession: □ Physician □ Nurse □ Other (Tech/Admin)

Experience: ____ years

Part 2: Measurement Items (Scale: 1 = Strongly Disagree, 2 = Disagree, 3 = Neutral, 4 = Agree, 5 = Strongly Agree)

**Code**	**Constructs & Items**	**1**	**2**	**3**	**4**	**5**
PU1	Using IoT technology improves my efficiency in managing medical assets (e.g., locating equipment).	□	□	□	□	□
PU2	IoT technology is useful for my daily job performance.	□	□	□	□	□
PU3	Using the IoT system enables me to accomplish asset management tasks more quickly.	□	□	□	□	□
PEU1	Learning to operate the IoT asset management system is easy for me.	□	□	□	□	□
PEU2	I find the interface of the IoT system clear and understandable.	□	□	□	□	□
PEU3	Becoming skillful at using the system does not require much effort.	□	□	□	□	□
TC1	The IoT system fits well with the way I currently work.	□	□	□	□	□
TC2	The IoT system is compatible with other existing information systems in the hospital.	□	□	□	□	□
TC3	Using the system does not require me to significantly change my work habits.	□	□	□	□	□
RC1	I must strictly adhere to data privacy and security regulations when using the system.	□	□	□	□	□
RC2	Hospital regulatory policies strictly limit how I use the system.	□	□	□	□	□
RC3	Complying with regulatory processes adds complexity to my use of the system.	□	□	□	□	□
OS1	The hospital provides necessary training for us to use the IoT system.	□	□	□	□	□
OS2	I have access to specialized technical support when the system encounters issues.	□	□	□	□	□
OS3	Hospital management encourages and supports our use of IoT technology at work.	□	□	□	□	□
ATU1	I have a positive attitude toward using the IoT system for asset management.	□	□	□	□	□
ATU2	I think using IoT technology in my work is a good idea.	□	□	□	□	□
ATU3	I like using the IoT system to assist with my work.	□	□	□	□	□
BI1	I intend to continue using the IoT system in the future.	□	□	□	□	□
BI2	I would recommend using the IoT system to my colleagues.	□	□	□	□	□
BI3	I will use the system in my work whenever possible.	□	□	□	□	□
AU1	I frequently use the IoT system in my daily work.	□	□	□	□	□
AU2	The IoT system has become an indispensable part of my work.	□	□	□	□	□
AU3	I use the various functions of the system skillfully and frequently.	□	□	□	□	□

## Appendix B. RC Entries and Legal Clauses Mapping Table

**Item**	**Item Wording**	**Primary Legal Anchor**	**Rationale**
RC1	I must strictly adhere to data privacy and security regulations when using the system.	PIPL Art. 5 (lawfulness, fairness, necessity) PIPL Art. 6 (purpose limitation) PIPL Art. 19 (minimisation, retention) PIPL Art. 28–29 (sensitive personal information incl. health data)	Captures the overarching legal obligation that clinicians internalise when interacting with any system handling patient-generated or patient-linked data. Health data is explicitly classified as sensitive personal information under PIPL, triggering the heightened protection regime that underlies much of the procedural burden.
RC2	Hospital regulatory policies strictly limit how I use the system.	DSL Art. 27 (data security management system required for data processors) PIPL Art. 51 (internal compliance management) PIPL Art. 52 (designation of personal information protection officer)	Captures the institutional translation of national laws into hospital-level operational rules. Under DSL Art. 27 and PIPL Arts. 51–52, hospitals must establish internal compliance management systems; these are the immediate source of the rule-environment that constrains clinician behaviour at the point of system use.
RC3	Complying with regulatory processes adds complexity to my use of the system.	PIPL Art. 55 (compliance audit obligation) PIPL Art. 52 (access logs and security monitoring) DSL Art. 29 (risk monitoring and incident response)	Captures the subjective procedural burden arising from the concrete implementation of the above obligations: multi-factor authentication, access logging, data masking steps, audit preparation, and incident-reporting workflows. This is the technostress-laden surface at which legal obligation meets clinical work and at which the burden reported in RC1 and RC2 materialises.
		Note. PIPL = Personal Information Protection Law of the People’s Republic of China (2021); DSL = Data Security Law of the People’s Republic of China (2021). Article numbers refer to the official promulgated text.	
